# A Review on Antibiotic Resistance: Alarm Bells are Ringing

**DOI:** 10.7759/cureus.1403

**Published:** 2017-06-28

**Authors:** Sojib Bin Zaman, Muhammed Awlad Hussain, Rachel Nye, Varshil Mehta, Kazi Taib Mamun, Naznin Hossain

**Affiliations:** 1 Maternal and Child Health Division, International Centre for Diarrhoeal Disease Research, Bangladesh; 2 Projahnmo Study Site, Johns Hopkin's University Bangladesh; 3 Health Consultant, Botane Skin Activates, Cape Town, South Africa; 4 Department of Internal Medicine, MGM Medical College, Navi Mumbai, India; 5 Nutrition and Clinical Services Division, International Centre for Diarrhoeal Disease Research, Bangladesh; 6 Department of Pharmacology, Dhaka Medical College, Dhaka, Bangladesh

**Keywords:** antibiotic resistance, knowledge, rational use

## Abstract

Antibiotics are the ‘wonder drugs’ to combat microbes. For decades, multiple varieties of antibiotics have not only been used for therapeutic purposes but practiced prophylactically across other industries such as agriculture and animal husbandry. Uncertainty has arisen, as microbes have become resistant to common antibiotics while the host remains unaware that antibiotic resistance has emerged. The aim of this review is to explore the origin, development, and the current state of antibiotic resistance, regulation, and challenges by examining available literature. We found that antibiotic resistance is increasing at an alarming rate. A growing list of infections i.e., pneumonia, tuberculosis, and gonorrhea are becoming harder and at times impossible to treat while antibiotics are becoming less effective. Antibiotic-resistant infections correlate with the level of antibiotic consumption. Non-judicial use of antibiotics is mostly responsible for making the microbes resistant. The antibiotic treatment repertoire for existing or emerging hard-to-treat multidrug-resistant bacterial infections is limited, resulting in high morbidity and mortality report. This review article reiterates the optimal use of antimicrobial medicines in human and animal health to reduce antibiotic resistance. Evidence from the literature suggests that the knowledge regarding antibiotic resistance in the population is still scarce. Therefore, the need of educating patients and the public is essential to fight against the antimicrobial resistance battle.

## Introduction and background

Antibiotics, either are cytotoxic or cytostatic to the micro-organisms, allowing the body’s natural defenses, such as the immune system, to eliminate them. They often act by inhibiting the synthesis of a bacterial cell, synthesis of proteins, deoxyribonucleic acid (DNA), ribonucleic acid (RNA), by a membrane disorganizing agent, or other specific actions [1]. Antibiotics may also enter the cell wall of the bacteria by binding to them, using the energy-dependent transport mechanisms in ribosomal sites, which subsequently leads to the inhibition of the protein synthesis [2].

To combat against infections or microbes, undoubtedly antibiotics are a blessing to human civilization that has saved millions of people [3]. Multiple varieties of the antibiotics have been used for therapeutic purposes over time. Antibiotics were seen as the ‘wonder drug’ in the mid-20th century. At the time, there was an optimistic belief that communicable disease was nearly coming to a complete halt. The beginning of modern “antibiotic era” was synonymously associated with two names Alexander Fleming and Paul Ehrlich [4]. Antibiotics were considered a magic bullet that selectively targeted microbes that were responsible for disease causation, but at the same time would not affect the host. Fleming was the first who cautioned about the potential resistance to penicillin if used too little or for a too short period of treatment [4]. The period from the 1950s to 1970s was thus considered as the golden era for the discovery of novel antibiotics classes [5].

Millions of metric tons of newer classes of antibiotics have been produced in last 60 years since its inception. Increased demand for antibiotics across many sectors has allowed for less expensive and off-label uses of drugs. Conversely, due to the enormous and irresponsible use of the antibiotics, has contributed significantly to the advent of the resistant strains [6]. In the previous days, the production of new antibiotics was directly proportional to the development of resistant strains. However, the mainstream approach in fighting against the diseases is now focused on the modification of existing antibiotics to combat emerging and re-emerging resistance of pathogens globally [5].

Resistance to an antibiotic develops in no time and hence, is a big matter of concern [7]. With the improvement of technology, more people are now aware of the ill-effects caused by resistance to the available drugs, however, very few take pro-active steps to curb the resistance by not over using the antibiotics [8]. In the developing world, almost all the antibiotics are available over the counter and can be bought without any medical prescription which is one of the most important factors in causing the resistance. Therefore, if the resistance to the antibiotics needs to be curbed, the only way shall be to educate the patients and the general public.

The present review is one such way to educate the public by showing the development and plausible future of antibiotic resistance and existing regulation to reduce the antibiotic resistance crisis.

## Review

Origin of antibiotic resistance

Antibiotic resistance was reported to occur when a drug loses its ability to inhibit bacterial growth effectively. Bacteria become ‘resistant’ and continue to multiply in the presence of therapeutic levels of the antibiotics [9]. Bacterias, when replicates even in the presence of the antibiotics, are called resistant bacterias.

**Figure 1:**
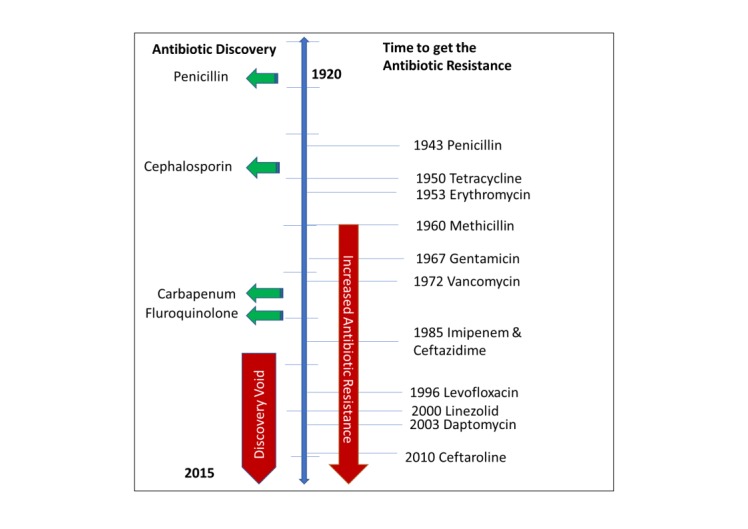
Graphical representation of onset of antibiotic resistance versus time to get antibiotic resistance History of antibiotic discovery (green arrow) and time of first reported year of antibiotic resistance (right side). The red arrow (lower direction) indicates the discovery void and increased antibiotic resistance. The blue line indicates the time flow

Antibiotics are usually effective against them, but when the microbes become less sensitive or resistant, it requires a higher than the normal concentration of the same drug to have an effect. The emergence of antimicrobial resistance was observed shortly after the introduction of new antimicrobial compounds [10]. Antibiotic resistance can occur as a natural selection process where nature empowers all bacteria with some degree of low-level resistance [3]. For example, one study confirmed that sulfamethoxazole and trimethoprim (TMP-SMZ), ampicillin and tetracycline that were commonly used in yesteryears, but now have no longer role in treating non-cholera diarrhea disease in Thailand [11]. At the same time, an another study conducted in Bangladesh showed the effectiveness of the same drugs in treating them effectively [12]. In fact, resistance was documented even before the beginning of the usage of the antibiotics in fighting the infection [13]. Non-judicial use of antibiotic is responsible for making microbes resistant. Since the introduction of sulfonamides in 1937, the development of specific mechanisms of resistance had provoked their therapeutic use. However, sulfonamide resistance was reported in the 1930s, which reveals the same mechanism of resistance that still operates even now, more than 80 years later [6]. Within six years of the production of the aminoglycosides, aminoglycoside-resistant strains of Staphylococcus aureus was developed [14]. Introduced in 1961, Methicillin was the first of the semisynthetic penicillinase-resistant penicillin to target strains of penicillinase-producing Staphylococcus aureus. However, resistance to methicillin was reported soon after its initiation [15]. Further, although fluoroquinolones were introduced for the treatment of Gram-negative bacterial diseases in the 1980s, fluoroquinolones resistance later revealed that these drugs were also used to treat Gram-positive infections [16]. Quinolone resistance emerged as a stepwise attainment of chromosomal mutations, particularly among the methicillin-resistant strains [Figure 1]. Most recently, the clinical isolates of Vancomycin-resistant Staphylococcus aureus (VRSA) were found in 2002, after 44 years of Vancomycin introduction to the market [17].

Antibiotics used in agriculture are often the same or similar to antibiotic compounds used clinically [18], this over-usage could also invite drug resistance. The food chain can be considered the main route of transmission of antibiotic-resistant bacteria between animal and human populations [19]. In some developed countries, animals receive antibiotics in their food, water, or parenterally which may be responsible for carrying microbe resistance to that specific antibiotic [18]. For example, the use of antibiotics in cattle feed as growth promoters increase antibiotic resistance [20]. Recent evidence suggests that poultry or pork might be a possible source of quinolone resistant-Escherichia coli in the rural villages in Barcelona, where one-fourth of children were found to be fecal carriers of these organisms. However, these kids were never exposed to quinolones [21].

Development of antibiotic resistance

Antibiotics fight to eliminate bacteria. Hence, bacteria tend to have a natural process that encourages resistance. The resistance process occurs via gene level mutations. [22]. Antibiotics induce selective pressure and the genes act in association with selective pressure [20]. Bacteria possess the quality to directly transfer genetic material between each other by transferring plasmids, which signifies that natural selection is not the only mechanism by which resistance evolves. Broad spectrum antibiotics are prescribed in hospitals as a solution for nosocomial infections; however, it increases resistance [16].

Antibiotics can generally eliminate the majority of bacteria in a colony. However, there may exist a different colony of bacteria that are genetically mutated which can lead to resistance [23]. The level of antibiotic-resistant infections was found to be strongly correlated with the degree of antibiotic consumption [24]. Development of resistance may also likely to occur if users fail to take their full course of prescribed antibiotic treatment. The bacteria subsequently remain untouched gaining more strength against the antibiotics [20]. Bacteria may collect multiple resistance traits over time and can become resistant to multiple classes of antibiotics [25]. For example, resistance was found in Staphylococci from the chromosomal mutations, ineffective transport of aminoglycosides into the bacteria as well as enzyme modification [16]. A single antibiotic may not only select resistance to one particular drug. Resistance can occur with other structurally related compounds of the same class. For example, resistance to tetracycline may incur resistance to oxytetracycline, chlortetracycline, doxycycline, and minocycline [26]. Antimicrobials possessed resistance genes that defend their antimicrobial products and these genes developed antibiotic resistance even long ago before the antibiotic started working for treatment purpose [27]. The different mechanisms of the common drug resistance are shown in Table 1.

**Table 1:** Table representing the mechanism of drug resistance of common antibiotics

Antibiotic class	Example(s)	Mode(s) of resistance
P-Lactams	Penicillins, Cephalosporins, Penems, Monobactams	Hydrolysis, efflux, altered target
Aminoglycosides	Gentamicin, Streptomycin, Spectinomycin	Phosphorylation, acetylation, nucleotidylation, efflux, altered target
Glycopeptides	Vancomycin, Teicoplanin	Reprogramming peptidoglycan biosynthesis
Tetracyclines	Minocycline, Tigecycline	Monooxygenation, efflux, altered target
Macrolides	Erythromycin, azithromycin	Hydrolysis, glycosylation, phosphorylation, efflux, altered target
Lincosamides	Clindamycin	Nucleotidylation, efflux, altered target
Streptogramins	Synercid	Carbon-Oxygen lyase, acetylation, efflux, altered target
Oxazolidinones	Linezolid	Efflux, altered target
Phenicols	Chloramphenicol	Acetylation, efflux, altered target
Quinolones	Ciprofloxacin	Acetylation, efflux, altered target
Pyrimidines	Trimethoprim	Efflux, altered target
Sulfonamides	Sulfamethoxazole	Efflux, altered target
Rifamycins	Rifampin	ADP-ribosylation, efflux, altered target
Lipopeptides	Daptomycin	Altered target
Cationic peptides	Colistin	Altered target, efflux

Consequence of antibiotic resistance

Antibiotic resistant organisms are known as superbugs. These are not only a laboratory concern but have become a global threat responsible for high death tolls and life-threatening infections [28]. Consequences of these infections are aggravated enormously in volatile situations such as civil unrest, violence, famine and natural disaster [29]. World Health Organization (WHO) [29] has warned that a post-antibiotic era will result in frequent infections and small injuries may result in death if we fail to act against antibiotic resistance. Multidrug-resistant bacteria causing more deaths worldwide. More than 63,000 patients from the United States of America (USA) die every year from hospital-acquired bacterial infections [30]. Every year, an estimated 25,000 patients die due to multiple drug resistance (MDR) bacterial infections in Europe [31]. Many countries are facing the burden of nosocomial *Staphylococcus aureus* (S. Aureus) infections as waves of clonal dissemination. Methicillin-resistant Staphylococcus aureus (MRSA) strains are rapidly spreading globally [16]. Estimated costs due to multidrug-resistant bacterial infection might result in extra healthcare costs and productivity losses [31]. It has been a standard practice for most of the pharmaceutical companies to distribute antibiotics that may no longer be effective or lacking regulatory approval [1]. Evidence shows that increased antibiotic use may result in a positive association with a higher prevalence of resistant microorganisms, while reduced antibiotic use showed lower resistance rates. There is clear evidence that patients historically treated with antibiotics are more likely to have antibiotic resistance [22]. Further, re-administration of antibiotics from the initial cycle accelerates resistance mechanisms [32]. Antibiotics encourage selective pressure for bacteria to evolve when administered frequently or irrationally. Individuals and states play a role in the evolution of antibiotic resistance [22]. For example, Clarithromycin consumption and its resistance similarly increased fourfold in Japan between 1993 and 2000 in comparison to other countries [33].

Regulatory issues related to antibiotic resistance

Congruent international management guidelines for daily antibiotic practices are yet unavailable. Hence, regulatory guidelines vary in different countries. Some countries have acted swiftly offering guidance e.g. United Kingdom, while other nations have yet to move toward interventions. The WHO has offered recommendations such as children in developing countries that antibiotics should only be used for the treatment of severe bloody diarrhea and cholera [34-35]. Since the beginning of the industrial revolution, we have dumped increase amounts of organic and inorganic toxins into streams, rivers, oceans, land, and air. In the personal care industry, there are insufficient guidelines for monitoring the home hygiene products which are likely to cause more risk for resistance because these products contain a high concentration of antibacterial ingredients [22].

With an abundance of evidence, there is no scope to ignore global antibiotic resistance. Antibiotic resistance can be more prevalent where antibiotic consumption is found to be higher. Lack of regulation and control in using antibiotics is prominent and needs to be targeted at a global capacity. Developing nations are at the greatest risk. Low prices of antibiotics, ease of availability and unnecessary use of antibiotics are causing more burden in developing countries [1]. Antibiotic use is relatively uncontrolled among the countries where there is no universal health coverage for its citizens [36]. Hence, irrational use of drugs has become a major concern. According to a study done in the United Kingdom, among the participants, 11.3% reported that they did not finish their last antibiotic course as prescribed. When asked about the reason why not comply with the course, 65% of the respondents stated that they felt better or forgot to take an antibiotic in time [37].

We are all affected by this multi-faceted public health issue. An all-encompassing problem that doesn’t just pertain to clinical personnel and microbiologists, but service personnel, industry stakeholders, specialists and the general public. We have to take necessary steps to tackle this complicated challenge. Social awareness, motivation, commitment in responsible sectors, stringent rules and regulation have to be prioritized. Further, we need the coupled action for the proper utilization of antibiotics, best management practices, and behavioral shifts across all industries that we can then combat against this public health burden. Application of modern technology can help the patient to take the antibiotic timely [38]. At present, the most notorious superbug is the Gram-positive organism Staphylococcus aureus [16]. This pathogen is frightening as its resistance to antibiotics is dramatically increasing. With an intimate history so closely tied to humans, Staphylococcus aureus is feared and at times, misunderstood [16]. These tendencies are causing higher resistance rates resulting in imminent hazards in human health. Notably, irrationality is observed in using antibiotics in livestock. Animals are given antibiotics for faster growth and disease prophylaxis. Strict and enforced regulations in the agricultural industry are needed to curb the harmful ripple effects.

Treatments for bacterial infections are becoming intensified every day. Infections remain as antibiotics gain resistance; treatment failure is common due to antibiotic resistance and multi-drug resistance, for e.g. tuberculosis. Newer and effective antibiotics that have no known resistance to bacteria are in high demand. Alternative treatment procedures are under consideration to fight bacterial infection. Passive immunization or administration of antibodies to non-immunized to prevent bacterial infections have been found effective [39]. Another effective intervention is phage therapy, whereas bacteriophages are used to treat pathogenic bacterial infections [40]. Many newer classes of antimicrobials to fight antibiotic resistance are in the pipeline for clinical trials [41]. Intervention strategies are aimed not only at targets but rather at the biological networks that may help to create new antibacterial therapies [42]. Combination therapies coupling antibiotics with antibiotic-enhancing phage have demonstrated the potential to be a promising antimicrobial intervention [4].

## Conclusions

Antibiotic resistance is at all time high in all the parts of the world. Despite measures taken by some member states of WHO, antibiotic use in humans, animals, and agriculture is increasing. The high economic burden in the healthcare sector has become a burning issue, due to extended hospital stays, isolation wards, stringent infection control measures and treatment failures. The public health leaders should establish a pan surveillance system coordinated at national and international levels, ongoing analysis and a mandatory reporting system for antibiotic resistance. Both domestic and global policies need to be conventional and adhered-to to stop the overuse and misuse of antibiotics.
